# Caring for homecare: A mixed methods analysis of the current and future role of home nurses

**DOI:** 10.1186/2049-3258-73-S1-P43

**Published:** 2015-09-17

**Authors:** Kristel De Vliegher, Anja Declercq, Bert Aertgeerts, Philip Moons

**Affiliations:** 1 Wit-Gele Kruis van Vlaanderen, Brussels, Belgium; 2 LUCAS, Leuven, Belgium; 3 ACHG, Leuven, Belgium; 4 Department of Public Health and Primary Care, Leuven, Belgium

## Introduction

The financial constraints and the shift of care from the hospital to the homecare setting challenges home nursing to provide care to sicker patients than in the past, to perform more intensive and technically complex nursing activities at home, and to think about a more efficient and effective use of the current home nursing staff. A mixed-method analysis was performed to understand the impact of these evolutions in home nursing in general and on the current and future role of home nurses.

## Aim

To understand the evolutions in home nursing and its impact on home nurses' activities.

## Method

A mixed-methods project was established comprising: (1) In-depth interviews with 15 home nurses and 8 medical specialists, and 2 focus groups with general practitioners; (2) Development and psychometric testing of a ‘24-hour recall instrument for home nursing’; (3) Quantitative measurement of the activities of 2478 home nurses; and (4) In-depth interviews with 12 home nurses, 12 healthcare assistants, and 8 managers of care.

## Results

(1) Technical activities are always performed in combination with more intellectual and psychosocial activities; (2) An instrument with 146 items, with a mean proportion of observed agreement of 0.94 and a mean kappa of 0.71; (3) The top 10 of activities performed is characterized by non-technical interventions; (4) Healthcare assistants can be an answer to the increased demand for care in home nursing.

